# Eye-Drops for Activation of DREADDs

**DOI:** 10.3389/fncir.2017.00093

**Published:** 2017-11-23

**Authors:** William T. Keenan, Diego C. Fernandez, Lukas J. Shumway, Haiqing Zhao, Samer Hattar

**Affiliations:** ^1^Biology Department, Johns Hopkins University, Baltimore, MD, United States; ^2^National Institute of Mental Health, National Institutes of Health, Bethesda, MD, United States; ^3^National Institute on Drug Abuse, National Institutes of Health, Baltimore, MD, United States

**Keywords:** chemogenetics, DREADD, behavior, neural circuit, clozapine-*N*-oxide

## Abstract

Designer Receptors Exclusively Activated by Designer Drugs (DREADDs) are an important tool for modulating and understanding neural circuits. Depending on the DREADD system used, DREADD-targeted neurons can be activated or repressed *in vivo* following a dose of the DREADD agonist clozapine-*N*-oxide (CNO). Because DREADD experiments often involve behavioral assays, the method of CNO delivery is important. Currently, the most common delivery method is intraperitoneal (IP) injection. IP injection is both a fast and reliable technique, but it is painful and stressful particularly when many injections are required. We sought an alternative CNO delivery paradigm, which would retain the speed and reliability of IP injections without being as invasive. Here, we show that CNO can be effectively delivered topically via eye-drops. Eye-drops robustly activated DREADD-expressing neurons in the brain and peripheral tissues and does so at the same dosages as IP injection. Eye-drops provide an easier, less invasive and less stressful method for activating DREADDs *in vivo*.

## Introduction

Scientists have developed a variety of methods to modulate targeted neuronal subpopulation *in vivo* to understand the neuronal circuits underlying behavior. Two such methods have become commonplace in modern neuroscience: chemogenetics and optogenetics. Both techniques rely on engineered proteins responsive to either chemical agonists in the case of chemogenetics or photons in the case of optogenetics.

A variety of chemogenetic tools have been developed over the past two decades, and have been recently reviewed (Sternson and Roth, 2014; Roth, 2016). However, a class named Designer Receptors Exclusively Activated by Designer Drugs (DREADDs) have emerged as the primary chemogenetic tool for modulation of specific cell types (Armbruster et al., 2007). The principal DREADDs used today are the activity enhancing G_q_-coupled hM3Dq receptor and the G_i_-coupled hM4D_i_ receptor (G_q_-DREADD and G_i_-DREADD) (Armbruster et al., 2007). DREADDs can be introduced virally or using an expanding collection of transgenic mice (Alexander et al., 2009; Guettier et al., 2009; Ray et al., 2011; Zhu et al., 2014, 2016). Both engineered receptors are activated following introduction of the chemical clozapine-*N*-oxide (CNO), and are being used to robustly modulate a variety of neuronal populations *in vivo* (Alexander et al., 2009; Atasoy et al., 2012; Garner et al., 2012; Keenan et al., 2016; Milosavljevic et al., 2016a). Additional CNO-responsive receptors have been engineered to activate G_s_ signaling (Guettier et al., 2009), arrestin signaling (Nakajima and Wess, 2012), and axon specific G_i_ signaling (Stachniak et al., 2014).

The primary advantage of the DREADD approach is that it can be used in live behaving animals without any need for complex equipment—optogenetics, for instance, requires delivering intense light to a particular brain region which often involves surgical implantation of an optical fiber. For *in vivo* studies using DREADDs, activation only requires delivery of CNO to the subject’s blood, which will eventually reach target neurons. While CNO is typically administered by intraperitoneal (IP) injection, it has also been delivered in drinking water (Jain et al., 2013) and by implanted minipumps (Donato et al., 2017). IP injection provides fine control over exact dosage and dose timing while drinking water and minipump approaches allow for chronic dosage without constant handling. However, each delivery method has disadvantages—IP injection causes both stress and pain which are undesirable when investigating animal behavior, particularly when studying aspects of behavior directly impacted by stress and pain. CNO in drinking water alleviates the confound of stress/pain but you lose control over precise dosage and dose timing. Additionally, increased costs associated with the large quantities of CNO required for dosing drinking water is also a limitation. The implanted minipump approach retains dosage control and allows for chronic administration, however this is achieved at the cost of requiring surgery and specialized equipment.

We sought to find and characterize a novel method of CNO delivery which alleviates some of the difficulties of currently used techniques. An often-used method of self-administration in humans is topical administration by eye-drops and subsequent absorption into the blood. Eye-drops are a non-invasive painless way of achieving precise dosage as well as dose timing. Eye-drop drug delivery has the added benefits of being exceptionally easy to perform as well as not requiring any additional equipment.

In this study, *we report that CNO can be delivered by eye-drop to activate DREADDs in vivo.* We first confirmed the capability of CNO eye-drops to activate DREADD-expressing neurons in the brain. Next, to investigate the feasibility of eye-drop CNO delivery *in vivo*, we utilized a subpopulation of retinal ganglion cells, intrinsically photosensitive retinal ganglion cells (ipRGCs), which drive robust and quantifiable pupil constriction when activated by light (Güler et al., 2008; Hatori et al., 2008; Chen et al., 2011) or DREADDs (Keenan et al., 2016; Milosavljevic et al., 2016b). Additionally, we determined the dose-response relationship and relative bioavailability of CNO delivered via eye-drop and IP injection.

## Materials and Methods

### Animal Husbandry

C57Bl/6 × Sv129 hybrid mice were used in all experiments. All mice were housed according to guidelines from the Animal Care and Use Committee of Johns Hopkins University. Male and female mice age 2–8 months were housed in plastic translucent cages with steel-lined lids in an open room. Ambient room temperature and humidity were monitored daily and tightly controlled. Food and water were available *ad libitum*. All mice were maintained in a 12 h:12 h light–dark cycle with light intensity around 100 lux.

### Drug Preparation

Clozapine-*N*-oxide (CNO, Sigma-Aldrich SKU:C0832-5MG) was dissolved in sterile 0.9% saline solution. CNO/saline solution was then diluted to achieve the dosage (mg/kg body weight) per mouse required for the experiment.

### CNO Delivery

Clozapine-*N*-oxide was delivered either by eye-drop or intraperitoneal injection. For eye-drops, CNO was diluted based on mouse weight to achieve the correct dose within a 1–2 μl dose. 1–2 μl was then loaded into P10 micropipette followed by immobilizing the mouse via scruff. The 1–2 μl range was chosen because it is large enough to be accurately pipetted and small enough to not drip off of the eye after application. The solution in the pipette was then expelled slowly so that a stable droplet forms on the pipette tip. The droplet was then carefully touched to the cornea of the mouse eye and the mouse was released. The pipette tip never contacts the mouse’s eye.

### Pupillometry

All mice were dark-adapted for at least 30 min prior to CNO delivery and subsequent pupil measurements. For all experiments, mice were unanesthetized and restrained by hand. Videos of the eye were taken using a Sony Handycam (FDR-AX33) mounted on a tripod a fixed distance from the mouse. Manual focus was maintained on the camera to ensure that only one focal plane existed for each mouse and that therefore variable distance from the camera should not contribute to differences in relative pupil area throughout the video. Pupil size was first recorded under dim red light and an external infrared light source to capture the dark-adapted baseline pupil size. CNO was then delivered as an eye-drop or injection and the mouse was returned to their cage. Pupil size was monitored at intervals described in the results section. All pupil images presented in the paper were cropped to a fixed square area surrounding the eye using GNU Image Manipulation Program (GIMP). The images were made grayscale and then brightness and contrast were adjusted to enhance visibility of the pupil and exported as PNG files.

### Data Analysis

Videos were transferred from the camera to a computer as Audio Video Interleave (AVI) files and individual frames were taken using VLC media player^1^ and saved in portable network graphics format (PNG). Pupil area was then quantified manually in ImageJ^2^ software. The pupil area was measured in pixels using the oval tool in which the four cardinal points of the oval were touching their respective edges of the pupil. The relative pupil area was calculated using Microsoft Excel in which the area during the stimulus was divided by the area prior to CNO dosage.

The dose-response curve was fit using a variable slope sigmoidal dose-response curve in Graphpad Prism 6. The top and bottom of the fit were constrained to 1.0 and between 0 and 0.10, respectively.

### Viral Infection

For viral infection of the retina, *Opn4*^Cre^ (Ecker et al., 2010) and littermate control mice were anesthetized by IP injection of avertin (2, 2, 2-Tribromoethanol) and placed under a stereo microscope. 1 μl of adeno-associated virus (AAV)2-hSyn-DIO-hM3DG_q_-mCherry (4.6 × 10^12^ viral particles/ml, Roth lab, UNC Vector Core) was placed on a piece of Parafilm and drawn into a 10-μl microcapillary tube (Sigma P0674) that had been pulled to a needle (Sutter Instruments, Model P-2000). The loaded needle was then placed in the holster of a pico-injector (Harvard Apparatus PLI-90). The needle punctured the eye posterior to the ora serrata and air pressure was used to drive the viral solution into the vitreous chamber of the eye to ensure delivery specifically to the retina. Mice recovered from surgery on a heating pad until they woke from anesthesia. All experiments were performed 3–5 weeks following viral injection.

For viral infection of the brain, mice were anesthetized by IP injection of avertin, and rAAV5-hSyn-hM3D(G_q_)-mCherry (3.4 × 10^12^ viral particles/ml, Roth lab, UNC Vector Core) was stereotaxically delivered. All coordinates used follow the Paxinos and Franklin atlas (Franklin and Paxinos, 1997). A 10-μl microcapillary pipette was pulled and loaded with the AAV solution. A total volume of 30 nl of AAV was injected using a microinjector (Nanojector II, Drummond Scientific Company). A heating pad was used to maintain the body temperature at ∼35°C. Before and after the surgery, systemic analgesics (buprenorphine, 0.1 mg/kg) were administrated.

### Immunofluorescence and Confocal Microscopy

Four weeks after brain injections, c-Fos induction (in AAV-infected neurons) was immunohistochemically evaluated in mice that were perfused 90 min after application of an eye-drop of CNO 0.001, 0.01, 0.1, and 1.0 mg/kg doses); mice were kept in constant darkness during the experiment. After perfusion, brains were post-fixed overnight, cryoprotected in 30% sucrose and subsequently sectioned on a cryostat (coronal sections, 40 μm). Brain sections were blocked for 2 h in PBS containing 0.3% Triton X-100 and 3% heat-inactivated goat serum and then incubated with a mouse IgG1 α-c-Fos (EnCor MCA-2H2; 1:500) overnight, at 4°C. After rinse, sections were incubated with goat anti-mouse IgG1 Alexa 488 (1:500) secondary antibody. Finally, slides were mounted in AntiFade medium (Molecular Probes), and images were acquired using a LSM-700 confocal microscope (Zeiss). Zeiss Zen software and ImageJ were used for subsequent file export.

All c-Fos staining was performed in parallel and images were taken with identical exposure. DREADD-mCherry expressing neurons co-labeled with c-Fos were quantified from six hippocampal slices at each CNO dose.

### Statistical Analysis

All statistical tests were performed in Graphpad Prism 6. Specific statistical comparisons are listed in the figure captions. Because the EC50 data appears to be a normal distribution on a log scale (log-normal distribution), all statistical tests and data analysis involving EC50 were performed on the log transformed data set.

## Results

### Eye-Drop CNO Activates DREADDs in the Brain

**FIGURE 1:**
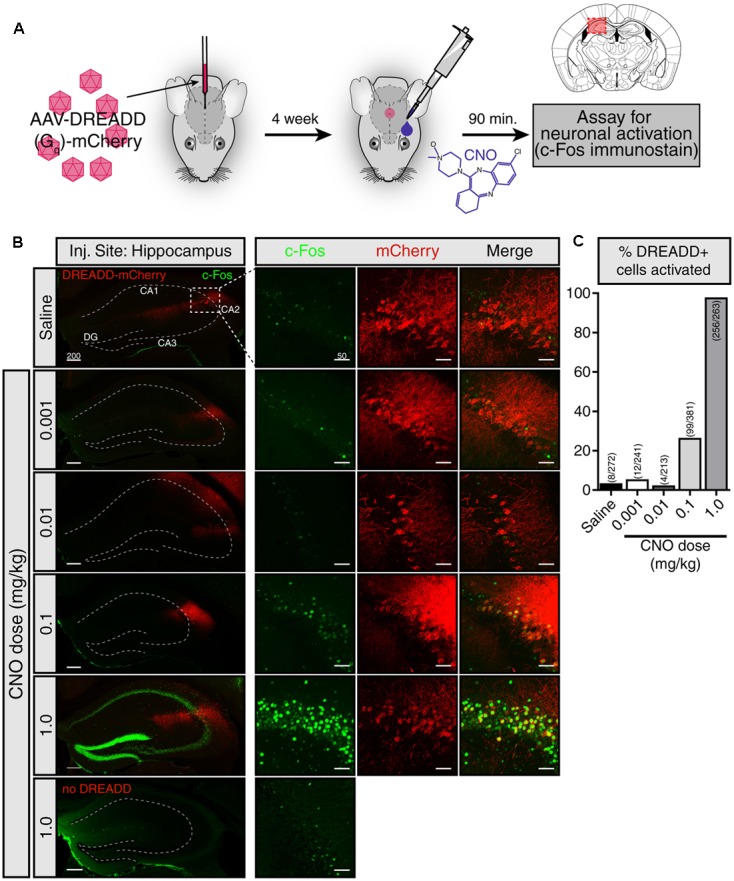
CNO eye-drops activate DREADDs in the brain. **(A)** Experimental approach. The effects of eye-drop delivery of clozapine-*N*-oxide (CNO) were evaluated in wild-type mice stereotaxically injected in the hippocampus with an AAV5-G_q_-DREADD-mCherry. Four weeks after AAV injection, a single eye-drop of CNO was administrated (1 μl, 0.001, 0.01, 0.1, and 1.0 mg/kg) and 90 min later c-Fos induction was evaluated. Mouse brain schematic with AAV injection site highlighted. **(B)** mCherry fluorescence (red) identifies DREADD-expressing neurons and c-Fos immunofluorescence (green) shows recently active cells. A saline eye-drop given to an infected mouse (top panel) as well as 1.0 mg/kg eye-drop to an uninfected mouse (bottom panel) are included as controls. (left) Low magnification view of the hippocampus including the viral injection site. Widespread c-Fos expression is visible only when 1.0 mg/kg CNO was delivered to a DREADD infected mouse. (right) Higher magnification view of the site of infection. Low levels of c-Fos expression were observed in the infected region in the mice given saline, 0.001 and 0.01 mg/kg CNO eye-drops. Higher c-Fos expression was present following 0.1 mg/kg CNO and drastically increased c-Fos throughout the hippocampus was observed following 1.0 mg/kg CNO. No elevation in c-Fos was observed in uninfected mice following a 1.0 mg/kg CNO eye-drop. **(C)** Clear colocalization of mCherry expression (red) and cFos staining (green), indicating activation of infected cells in response to CNO, was quantified by hand in six hippocampal slices in each condition. Saline: 8 of 272 cells (2.9%); 0.001 mg/kg CNO: 12 of 241 (4.98%); 0.01 mg/kg: 4 of 213 (1.88%); 0.1 mg/kg: 99 of 381 (25.99%); 1.0 mg/kg: 256 of 263 (97.34%). All c-Fos staining was performed in parallel and images were taken with identical exposure. Scale bars: left = 200 μm; right = 50 μmDG: dentate gyrus.

We first sought to evaluate whether CNO eye-drops effectively activate DREADDs in the brain. To do so, we injected a G_q_-DREADD-containing virus [AAV5-hSyn-hM3D(G_q_)-mCherry] into a the hippocampus (**Figure 1A**). Four weeks following viral injection, we applied an eye-drop of saline or CNO (0.001, 0.01, 0.1, and 1.0 mg/kg doses) and evaluated neuronal activation 90 min afterward by assaying the expression of the immediate early-gene c-Fos. Using the DREADD-mCherry fusion protein to identify virally infected cells, we observed robust c-Fos staining following 1.0 mg/kg CNO in neurons expressing the DREADD-mCherry reporter, indicating that the CNO eye-drop successfully activated G_q_-DREADD in those cells (**Figure 1B**). DREADD activation by 1.0 mg/kg CNO in the subpopulation of infected hippocampal neurons resulted in widespread activation and c-Fos induction of the entire hippocampus. Less intense c-Fos induction was observed following 0.1 mg/kg CNO and no obvious c-Fos induction followed 0.01 and 0.001 mg/kg CNO. A saline eye-drop or 1.0 mg/kg CNO dose applied to a non-infected mouse did not elicit hippocampal c-Fos expression (**Figure 1B**). Finally, we quantified the percentage of DREADD-mCherry+ cells clearly expressing c-Fos following saline and each dose of CNO [Saline: 8 of 272 cells (2.9%); 0.001 mg/kg CNO: 12 of 241 (4.98%); 0.01 mg/kg: 4 of 213 (1.88%); 0.1 mg/kg: 99 of 381 (25.99%); 1.0 mg/kg: 256 of 263 (97.34%)] (**Figure 1C**). 1.0 mg/kg CNO appears to be an effective dose for robust activation of DREADD-expressing neurons in the brain. This result confirms the ability of CNO delivered by eye-drop to enter the blood and activate DREADDs in the brain.

### Eye-Drop and IP Injection Evoked Responses Have Similar Dose Efficiency

To visualize and quantify DREADD activity *in vivo*, we utilized a genetically defined subpopulation of melanopsin expressing retinal ganglion cells, intrinsically photosensitive retinal ganglion cells ipRGCs (ipRGCs), which we and others have shown to drive pupil constriction when activated by G_q_-DREADD (Keenan et al., 2016; Milosavljevic et al., 2016b). This system gives us a readily observable and easily quantifiable output of DREADD activation *in vivo*, allowing us to quantitatively compare the effectiveness of eye-drops and IP injection in real-time The *Opn4*^Cre^ (*Opn4* gene codes melanopsin) mouse (Ecker et al., 2010) provides us genetic access to ipRGCs, and a Cre-dependent G_q_-DREADD AAV allows us to express G_q_-DREADD specifically in these cells.

**FIGURE 2:**
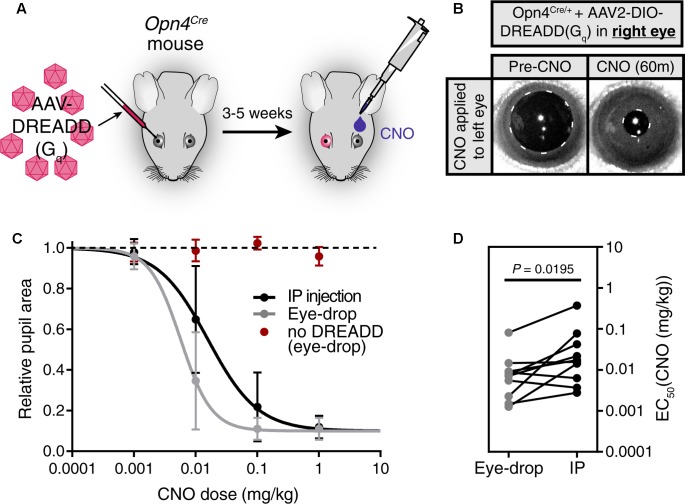
Eye-drop administration of CNO activates DREADDs *in vivo* similar to IP. **(A)** Experimental approach. AAV carrying a Cre-dependent G_q_-DREADD was injected into the right eye of mice with Cre expression in ipRGCs (*Opn4*^Cre^ mice). 3–5 weeks later, CNO was administered by eye-drop to the left eye or by IP injection. **(B)** DREADD activation is visualized by measuring pupil constriction in response to 1 μl CNO (1 mg/kg). (left) Baseline pupil size before CNO. (right) Pupil constriction observed in response to CNO applied directly to the left uninfected eye. **(C)** Dose-response curves for CNO applied via IP injection or eye-drop. Four doses were administered: 0.001, 0.01, 0.1, and 1.0 mg/kg. Data fit with a sigmoidal curve (*n* = 10, mean ± SD). Additionally, CNO eye-drops were administered to mice lacking DREADD virus injection (*n* = 6, mean ± SD). **(D)** CNO dose required for half-maximal constriction (EC_50_) determined for both eye-drop and IP injection. EC_50_ extracted from the sigmoidal curve fits for each mouse (points are individual mice, lines connect EC_50_ values for the same mouse). Statistical significance determined by a non-parametric Wilcoxon matched-pairs signed ranked test (*P* = 0.0195).

We first injected an AAV carrying a Cre-dependent G_q_-DREADD construct (AAV2-hSyn-DIO-hM3DG_q_-mCherry) into only the right eye of *Opn4*^Cre^ mice, leaving the left eye uninfected (**Figure 2A**). 3–5 weeks after infection, we applied a 1 μl CNO (0.1 mg/kg) eye-drop to the uninfected left eye. We observed robust pupil constriction (**Figure 2B**) as has been observed previously in response to IP CNO (Keenan et al., 2016; Milosavljevic et al., 2016b). This result further demonstrates that CNO delivered via eye-drop is absorbed into the blood and delivered to distant tissues at working concentrations *in vivo*.

After confirming the feasibility of eye-drops as a delivery method, we next compared the CNO doses required to elicit responses when using eye-drop or IP delivery (**Figures 2C,D**). To do so, we administered doses of 0.001, 0.01, 0.1, and 1.0 mg/kg CNO via eye-drop or IP injection and monitored pupil constriction (**Figure 2C**, *n* = 10). We observed similar dose responses for both methods, with eye-drops displaying a small but statistically significant decrease in the dose required to achieve half-maximal response (EC_50_) (**Figure 2D**, *P* = 0.0195 by Wilcoxon matched-pairs signed rank test). This difference could be explained by changes in blood absorption efficiency or potentially by reduced stress responses in these mice after eye-drop as opposed to IP injection. However, the magnitude of the difference is minor and essentially irrelevant when considering the practical application of either technique.

## Discussion

We have shown that eye-drops are an effective way to deliver CNO and activate DREADDs for *in vivo* studies. Eye-drops offer an alternative to current CNO delivery methods: drinking water and IP injection. When chronic DREADD activation is necessary and dose timing is unimportant, drinking water still provides the best dosing method. However, in the majority of DREADD experiments in which IP injection would be used, our method provides several advantages: (1) ease of application, (2) non-invasive, (3) less pain and stress, (4) cost/waste reduction (no syringes).

We hope that the widespread use of eye-drops in the place of IP injection will further simplify performing DREADD experiments and significantly reduce the distress inflicted on test subjects during *in vivo* experimentation.

## Ethics Statement

Animal experimentation: this study was performed in strict accordance with the recommendations in the Guide for the Care and Use of Laboratory Animals of the National Institutes of Health. All mice were housed according to guidelines from the Animal Care and Use Committee of Johns Hopkins University (Protocol # MO16A212), and used protocols approved by the JHU animal care and use committee.

## Author Contributions

WK conceived of, designed and performed experiments, and wrote the manuscript. DF performed experiments, contributed figures and edited the manuscript. LS performed experiments and edited the manuscript. HZ and SH contributed to experimental design and edited the manuscript.

## Conflict of Interest Statement

The authors declare that the research was conducted in the absence of any commercial or financial relationships that could be construed as a potential conflict of interest. The reviewer RDL and handling Editor declared their shared affiliation, and the handling Editor states that the process nevertheless met the standards of a fair and objective review.

## Footnotes

1 http://www.videolan.org/vlc/

2 http://rsbweb.nih.gov/ij/
